# Antibiotic Prescription in Dentistry: Trends, Patient Demographics, and Drug Preferences in Germany

**DOI:** 10.3390/antibiotics14070676

**Published:** 2025-07-03

**Authors:** Lisa Lotta Cirkel, Jens Martin Herrmann, Claudia Ringel, Bernd Wöstmann, Karel Kostev

**Affiliations:** 1 Department of Cariology, Periodontology, and Endodontology, Dental Clinic, Justus Liebig University, 35392 Giessen, Germany; 2 Real World Solutions, IQVIA, 04229 Leipzig, Germany; 3 Department of Prosthodontics, Dental Clinic, Justus Liebig University, 35392 Giessen, Germany; 4 Epidemiology, IQVIA, 60549 Frankfurt, Germany

**Keywords:** dental antibiotic prescription, antimicrobial resistance, amoxicillin, clindamycin, prescription trends, dental public health, odontogenic infections, COVID-19, Germany

## Abstract

**Background and objectives:** ABs are widely used in dental practice in the treatment of odontogenic infections and as systemic prophylaxis in high-risk patients. However, AB overuse contributes to antimicrobial resistance (AMR), which is a major global concern. This study examined dental AB prescribing trends in Germany in 2024, focusing on the share of overall AB prescriptions, patient demographics, and commonly used agents. **Methods:** This retrospective cross-sectional study used data from the IQVIA Longitudinal Prescription Database (LRx), covering approximately 80% of prescriptions reimbursed by statutory health insurance funds in Germany. Patients with at least one AB prescription (ATC code: J01) issued by a dentist in 2024 were analyzed. Descriptive statistics covered age, sex, and prescribed substances. **Results:** In 2024, German dentists prescribed ABs to 2,325,500 patients, accounting for 13.9% of all patients in the database who received AB prescriptions. Dentists were the second-largest group of AB prescribers, surpassed only by general physicians. Amoxicillin (54.2%) was most frequently prescribed, followed by amoxicillin with clavulanic acid (24.5%) and clindamycin (21.0%). Dental patients receiving AB prescriptions were older (mean age: 49.8 years) than the general antibiotic patient population (44.7 years). Interestingly, dental AB prescriptions increased during the COVID-19 pandemic, in contrast to the sharp overall decline in AB prescriptions. Between 2015 and 2019, the proportion of dental antibiotic prescriptions showed a moderate upward tendency, followed by a marked increase during the COVID-19 pandemic and a subsequent decline. In contrast, the number of patients receiving antibiotic prescriptions from other medical disciplines decreased over the same period. One particularly notable finding was the extended use of clindamycin, a reserve AB with known side effects and resistance risks, in dentistry. **Conclusions:** Dentists are responsible for a significant share of AB prescriptions in Germany. The rise in dental AB prescriptions, particularly the frequent prescription of clindamycin, underscores the need for interventions such as updated clinical guidelines and awareness campaigns concerning AB-related risks and their mitigation directed at dentists. These could focus on microbial culture and sensitivity testing and patient adherence education and control for targeted AB interventions. Emphasizing preventive and alternative anti-infective treatment strategies in dentistry may also help to contain AMR.

## 1. Introduction

Antibiotics are a standard treatment option in current medical practice. They are often prescribed in dentistry for prophylaxis in high-risk patients and for treating odontogenic infections—bacterial inflammations that originate in the teeth or periodontium [1].

About 10% of all antibiotic prescriptions in industrialized countries are for dental purposes [2,3]. While overall counts of antibiotic prescriptions fell by 41.6% between 2012 and 2021, the proportion of dental prescriptions increased [4].

Antimicrobial resistance (AMR) is a rising concern for the medical community and includes resistance observed in bacteria causing odontogenic infections [5]. Estimates from 2019 indicate that approximately 1.27 million deaths worldwide are directly attributable to resistant bacterial infections, with up to 4.95 million deaths associated with bacterial AMR [6]. Given the global rise in AMR, it is considered a major public health challenge and the rational use of antibiotic medications is coming under increasing scrutiny.

In Germany, dentists account for a substantial proportion of all antibiotic prescriptions. Among the most commonly prescribed antibiotics in dental practice are amoxicillin (sometimes in combination with clavulanic acid), clindamycin, and penicillin [2]. Studies in Germany show that certain oral bacterial strains already exhibit a considerable degree of resistance to commonly used antibiotics. The most frequently detected aerobic bacteria in odontogenic infections include *Viridans streptococci* and *Staphylococcus aureus* [5]. Despite comparatively high resistance rates in clinically relevant pathogens such as *Streptococcus* spp. and *Staphylococcus aureus* (17–19%), studies show that clindamycin continues to be prescribed often [2,5,7]. The resistance rates for penicillin and for amoxicillin in combination with clavulanic acid are significantly lower [5,8].

In view of the prescribing behavior outlined above, it is clear that dentistry professionals may play a significant role in influencing antibiotic resistance, and the importance of indication- and guideline-based antibiotic therapy across all medical disciplines must not be underestimated.

The aim of this analysis was to examine the antibiotic prescribing behavior and trends of German dentists in 2024 and over time since 2015. The study focused on the development of the proportion of prescriptions issued by dentists compared to the total number of antibiotic prescriptions issued, the demographic distribution of patients, and preferred active substances in dental antibiotic regimens.

## 2. Results

A total of 16,713,894 patients in the LRx database received at least one antibiotic prescription in 2024, corresponding to approximately 18.7 million patients in Germany with statutory health insurance. Dentists prescribed antibiotics to 2,325,500 patients, accounting for 13.9% of all patients in the database who had received antibiotic prescriptions. As such, German dentists rank second after primary care physicians (i.e., general practitioners or physicians specializing in internal medicine, 66.8%).

While the overall number of antibiotic prescriptions declined in 2020 and 2021 during the COVID-19 pandemic, the number of antibiotic prescriptions issued by dentists remained relatively stable before decreasing slightly in 2022 and 2023. Despite an increase in Germany’s population (from 81.7 million in 2015 to 83.6 million in 2024), the total number of patients receiving antibiotics declined from 18.5 million in 2015 to 16.7 million in 2024. However, the number of antibiotic prescriptions issued by dentists increased significantly from 2,256,408 (12.1% of all antibiotic recipients) in 2015 to 2,325,500 (13.9%) in 2024 (Table 1).

**Figure 1:**
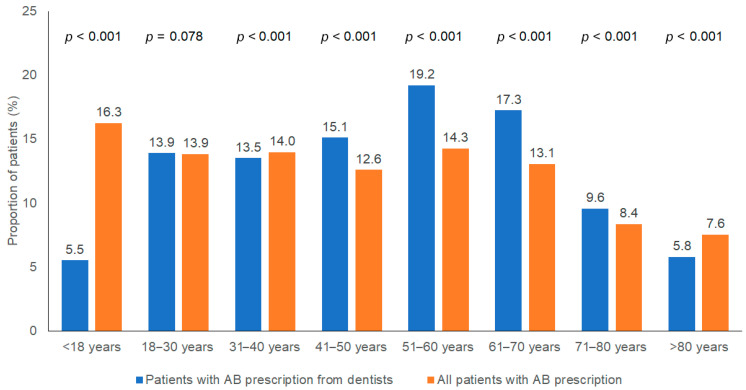
Age structure of antibiotic users who were prescribed antibiotics by dentists and in the total population.

The average age of antibiotic users receiving their prescriptions from dentists was 49.8 years (SD: 19.8), which was higher than that of the overall antibiotic user population (44.7 years; SD: 23.0) (*p* < 0.001). Figure 1 illustrates the age distribution of antibiotic users whose prescriptions came from dentists compared to all antibiotic users. Among antibiotic users whose prescriptions were issued by dentists, the most common age groups were 51–60 years (19.2%), 61–70 years (17.3%), and 41–50 years (15.1%). By contrast, the most common age group among all antibiotic users was under 18 years (16.3%), while only 5.5% of dentist-prescribed antibiotics were in this cohort (Figure 1).

In 2024, some 13.9% of all antibiotic users received antibiotic prescriptions from dentists; however, the proportion was higher among older age groups—18.7% in patients aged 51–60 years and 18.3% in those aged 61–70 years compared to just 4.7% among children and adolescents (*p* < 0.001).

The proportion of female patients was 53.1% among users of antibiotics prescribed by dentists, which was slightly lower than the overall antibiotic user population (56.9%) (*p* < 0.001). Table 2 presents the distribution of different antibiotic drugs prescribed by dentists and by all physicians. The antibiotic most frequently prescribed by dentists was amoxicillin (54.2%), followed by the fixed-dose combination of amoxicillin and clavulanic acid (24.5%) and then clindamycin (21.0%). The proportions of other antibiotics were negligible, with each accounting for less than 1% of prescriptions. In the overall antibiotic user population, amoxicillin (27.2%) and amoxicillin + clavulanic acid (19.9%) were the most frequently prescribed antibiotics, albeit at lower proportions than in dentist prescriptions. Clindamycin was less commonly prescribed outside of dentistry, whereas azithromycin and cefuroxime axetil were more frequently used.

Notably, dentists accounted for 27.8% of all amoxicillin prescriptions, 17.2% of all amoxicillin + clavulanic acid prescriptions, and 42.4% of all clindamycin prescriptions in 2024.

## 3. Discussion

In 2024, dentists in Germany ranked second (13.9%) after general practitioners (66.8%) in terms of the proportion of antibiotic prescriptions issued. While the total number of antibiotic prescriptions declined between 2015 and 2024, the proportion of prescriptions issued by dentists increased during the same period.

One notable finding of our study is the relative increase in antibiotic prescriptions in dentistry during the COVID-19 pandemic, despite a general decline in total prescriptions. Compared to the gradually increasing trend observed between 2015 and 2019, the proportions of dental antibiotic prescriptions in 2020 (17.9%) and 2021 (18.1%) were markedly elevated. This pattern likely reflects the influence of the COVID-19 pandemic, during which access to dental care was temporarily limited and both patients and providers had to first adapt to the new situation and perceived infection risks, which may have influenced treatment decisions. Comparable developments occurred internationally. Studies carried out in England and Norway report an increase in dental antibiotic prescriptions during the pandemic [9,10], while a recent study from the US shows similar developments. In the US, the rate of antibiotic prescriptions issued by general dentists remained stable from 2018 to 2022, although the total number of all antibiotic prescriptions declined by 5.4% during the same period. As a result, the relative share of dental prescriptions increased, particularly between 2020 and 2022 during the COVID-19 pandemic [11]. One possible explanation is the limited dental care available during the pandemic and the reluctance of many patients to visit dental practices, e.g., due to fear of infection through aerosols [9,12]. As a result, check-ups and treatment appointments were often postponed, which may have led to the increased use of antibiotic prescriptions in acute cases as a substitute for interventional treatments. While antibiotic prescribing declined overall across medical disciplines, this trend was less pronounced in dental care. One potential factor contributing to this pattern is the increasing lifespan and the higher proportion of elderly people in the German population. As the prevalence of periodontitis increases with age [13], the demand for adjunctive antibiotic therapy during periodontal treatment may also rise as a result.

Our data show that dentists mainly prescribe antibiotics to middle-aged and older patients, while children and adolescents are rarely affected. This contrasts with other medical specialties, where young adolescents receive antibiotics more frequently. The difference could be due to the German health programs for prophylaxis in children and adolescents, which are based on preventive measures such as regular dental check-ups, fluoride applications and individual prophylaxis programs that are available to all children under the statutory health insurance. In addition, comorbid diseases appear to occur less frequently in children, which could also contribute to the lower frequency of antibiotic prescriptions in this age group.

Antibiotic prescriptions issued in dental practices in 2024 primarily included one or a combination of three active ingredients: amoxicillin, amoxicillin in combination with clavulanic acid, and clindamycin. While amoxicillin and its combination are also widely used in other medical fields, the above-average use of clindamycin by dentists is striking. Clindamycin was the third most prescribed antibiotic by dentists in Germany in 2024. It is often used as an alternative in patients with penicillin allergy and is associated with a known side effect profile, such as the risk of pseudomembranous colitis, and a documented high resistance rate in pathogens causing odontogenic infections [5,14]. Amoxicillin and clindamycin also dominated dental prescribing patterns in the US. Between 2018 and 2022, the proportion of amoxicillin increased, while that of clindamycin declined—a decrease that may be due to the ADA (American Dental Association) guideline introduced in 2019. This guideline recommends amoxicillin as a first-line therapy and advises against the routine use of clindamycin, particularly due to its known side effect profile [11]. By contrast, the proportion of clindamycin in Germany remains high at 21.0%.

Published in 2016 and still serving as the primary reference in clinical practice despite being formally expired, the Odontogenic Infections Guideline recommends clindamycin for patients with penicillin allergies [5]. The results of a recent study show that 8–15% of the population in the US report a penicillin allergy, but up to 95% of these cases do not display a true allergic reaction in tests [15]. These (undiagnosed or) misdiagnosed allergies lead to the introduction of alternative antibiotics, such as clindamycin. Another indication for dental antibiotic therapy is the risk of endocarditis. Updated in 2021, the American Heart Association (AHA) guidelines for the prevention of infectious endocarditis no longer recommend the use of clindamycin as a prophylaxis before dental procedures. This decision is based on the increased risk of serious side effects, particularly *Clostridioides difficile* infections, compared to other antibiotics. Instead, alternative agents such as cephalexin, azithromycin, clarithromycin, or doxycycline are recommended for patients with penicillin allergies [16]. The recommendations of the German Society of Dentistry and Oral Medicine [17] still apply in Germany; although they are no longer up to date, they are considered the authoritative reference in clinical practice. It is anticipated that the new, well-founded AHA guideline will soon be officially adapted in Germany.

This study is subject to several limitations associated with the use of prescription data. The dataset does not include diagnosis codes or clinical context information, which limits the assessment of the medical justification for the use of antibiotics. Furthermore, the data provide no information on possible side effects. These limitations could restrict the interpretation of the results. Nevertheless, they are offset by the key strengths of the study: the large number of patients; the long observation period; and the nationally representative prescription data provide a solid basis for analyzing dental prescribing behavior.

These findings emphasize the substantial proportion of outpatient antibiotic prescriptions that are issued by dental practitioners and their potential impact on antimicrobial resistance patterns. Although national and international guidelines for antibiotic use in dentistry are in place, the extent to which they are consistently followed in clinical practice remains uncertain. Although this study reports the distribution of substances prescribed by dentists in 2024, further research should examine temporal trends in the use of individual antibiotic agents, particularly amoxicillin and clindamycin, to assess whether guideline adherence has improved over time. Future research should examine dentists’ awareness and execution of microbial culture and sensitivity testing for targeted antibiotic therapies, resistance mechanisms, and the safety profiles of frequently used agents. On the other hand, an increased emphasis on dentist–patient communication and direct-to-patient educational campaigns about the increased risk of AMR through non-adherence to or persistence with antibiotic therapies will be critical to our efforts to contain AMR. Finally, targeted regimen strategies—including updated guideline dissemination, educational initiatives, and prescription audits—could support more rational and controlled antibiotic use in dental care. A special focus should also be placed on further training content in the dental field that addresses the responsible use of antibiotics, as every practicing dentist in Germany is legally obliged to undergo regular and individual further training. These measures may help to reduce unnecessary prescriptions and mitigate the broader development of antimicrobial resistances.

## 4. Materials and Methods

Database: this retrospective cross-sectional study utilized the IQVIA Longitudinal Prescription Database (LRx) [18]. The database covers approximately 80% of prescriptions reimbursed by statutory health insurance funds in Germany. Data are available at the patient level, including information on the patient age, sex, and the specialties of prescribing physicians. All patient data are fully blinded (anonymized) in accordance with data privacy regulations. Each recorded prescription includes comprehensive product details (e.g., brand name, active substance, package size, and product form) as well as dispensing dates. However, the database does not contain information on diagnoses, laboratory tests, or procedures [18]. This database has been widely used in previous pharmacoepidemiological studies [19,20].

Study population and outcomes: this retrospective cohort study included patients who received at least one antibiotic prescription (ATC: J01) from a dentist in Germany in December 2024. The study measured time trends in antibiotic prescriptions by dentists (comparing 2024 to 2015 in the same database), age and sex distribution of users of antibiotics prescribed by dentists, proportion of overall antibiotic prescriptions issued by dentists, stratified by age group and sex, and proportions of different antibiotic drugs prescribed by dentists.

Statistical analyses: descriptive analyses were conducted, and *p*-values from the Chi-squared test were reported to assess differences in age group distribution between patients receiving AB prescriptions from dentists and all patients with AB prescriptions. However, due to the large sample size, even small differences can result in highly significant *p*-values.

All analyses were conducted using SAS version 9.4 (SAS Institute, Cary, NC, USA).

## Figures and Tables

**Table 1:** Patients with antibiotic prescription overall and those prescribed antibiotics by dentists from 2015 to 2024 (absolute numbers).

Year	All Patients with AB Prescription	Patients with AB Prescription from Dentists	AB Prescription by Dentist (%), 95% Confidence Intervals
2015	18,498,114	2,256,408	12.2% [12.1–12.2]
2016	18,330,608	2,286,556	12.5% [12.5–12.5]
2017	18,056,249	2,371,424	13.1% [13.1–13.2]
2018	17,536,447	2,314,025	13.2% [13.2–13.2]
2019	16,481,120	2,332,442	14.2% [14.1–14.2]
2020	12,803,231	2,287,982	17.9% [17.9–17.9]
2021	11,632,986	2,108,601	18.1% [18.1–18.2]
2022	14,303,669	1,758,852	12.3% [12.3–12.3]
2023	16,217,280	1,667,707	10.3% [10.3–10.3]
2024	16,713,894	2,325,500	13.9% [13.9–13.9]

**Table 2:** Proportion of antibiotic drugs prescribed in total and by dentists in 2024.

Drug	Patients with AB Prescription from Dentists (N, %)	All Patients with AB Prescription (N, %)
Amoxicillin	1,261,403 (54.2)	4,541,188 (27.2)
Amoxicillin + clavulanic acid	570,621 (24.5)	3,319,549 (19.9)
Clindamycin	488,606 (21.0)	1,153,309 (6.9)

## Data Availability

Data were obtained from IQVIA and are available upon reasonable request with the permission of IQVIA. Restrictions apply due to data protection requirements.
